# AmyloGraph: a comprehensive database of amyloid–amyloid interactions

**DOI:** 10.1093/nar/gkac882

**Published:** 2022-10-16

**Authors:** Michał Burdukiewicz, Dominik Rafacz, Agnieszka Barbach, Katarzyna Hubicka, Laura Bąkała, Anna Lassota, Jakub Stecko, Natalia Szymańska, Jakub W Wojciechowski, Dominika Kozakiewicz, Natalia Szulc, Jarosław Chilimoniuk, Izabela Jęśkowiak, Marlena Gąsior-Głogowska, Małgorzata Kotulska

**Affiliations:** Institute of Biotechnology and Biomedicine, Autonomous University of Barcelona, Campus Universitat Autònoma de Barcelona Plaça Cívica Bellaterra, s/n, 08193 Cerdanyola del Vallès, Barcelona, Spain; Clinical Research Centre, Medical University of Białystok, Kilińskiego 1, 15-369 Białystok, Poland; Faculty of Mathematics and Information Science, Warsaw University of Technology, Koszykowa 75, 00-662 Warsaw, Poland; Department of Biomedical Engineering, Faculty of Fundamental Problems of Technology, Wrocław University of Science and Technology, Wybrzeże Wyspiańskiego 27, 50-370 Wrocław, Poland; Department of Biomedical Engineering, Faculty of Fundamental Problems of Technology, Wrocław University of Science and Technology, Wybrzeże Wyspiańskiego 27, 50-370 Wrocław, Poland; Faculty of Mathematics and Information Science, Warsaw University of Technology, Koszykowa 75, 00-662 Warsaw, Poland; School of Biosciences, College of Life and Environmental Sciences, University of Birmingham, Edgbaston, Birmingham B15 2TT, United Kingdom; Faculty of Medicine, Wrocław Medical University, Ludwika Pasteura 1, 50-367 Wrocław, Poland; Faculty of Medicine, Wrocław Medical University, Ludwika Pasteura 1, 50-367 Wrocław, Poland; Department of Biomedical Engineering, Faculty of Fundamental Problems of Technology, Wrocław University of Science and Technology, Wybrzeże Wyspiańskiego 27, 50-370 Wrocław, Poland; Laboratory of Microbiome Immunobiology, Hirszfeld Institute of Immunology and Experimental Therapy, Polish Academy of Sciences, Weigla 12, 53-114 Wrocław, Poland; Department of Biomedical Engineering, Faculty of Fundamental Problems of Technology, Wrocław University of Science and Technology, Wybrzeże Wyspiańskiego 27, 50-370 Wrocław, Poland; Department of Genomics, Faculty of Biotechnology, University of Wrocław, Fryderyka Joliot-Curie 14a, 50-383 Wrocław, Poland; Department of Pharmacology, Wroclaw Medical University, Mikulicza-Radeckiego 2, 50-345 Wrocław, Poland; Department of Biomedical Engineering, Faculty of Fundamental Problems of Technology, Wrocław University of Science and Technology, Wybrzeże Wyspiańskiego 27, 50-370 Wrocław, Poland; Department of Biomedical Engineering, Faculty of Fundamental Problems of Technology, Wrocław University of Science and Technology, Wybrzeże Wyspiańskiego 27, 50-370 Wrocław, Poland

## Abstract

Information about the impact of interactions between amyloid proteins on their fibrillization propensity is scattered among many experimental articles and presented in unstructured form. We manually curated information located in almost 200 publications (selected out of 562 initially considered), obtaining details of 883 experimentally studied interactions between 46 amyloid proteins or peptides. We also proposed a novel standardized terminology for the description of amyloid–amyloid interactions, which is included in our database, covering all currently known types of such a cross-talk, including inhibition of fibrillization, cross-seeding and other phenomena. The new approach allows for more specific studies on amyloids and their interactions, by providing very well-defined data. AmyloGraph, an online database presenting information on amyloid–amyloid interactions, is available at (http://AmyloGraph.com/). Its functionalities are also accessible as the R package (https://github.com/KotulskaLab/AmyloGraph). AmyloGraph is the only publicly available repository for experimentally determined amyloid–amyloid interactions.

## INTRODUCTION

Amyloids are proteins able to self-assembly into insoluble β-sheet supra-molecular fibrils characterized by very regular beta-cross structures. Some of them interact with each other during fibrillization, which may accelerate or slow down development of fibrils or even lead to the formation of heterogeneous fibrils (1). Interactions between amyloid proteins raise a growing interest since they may contribute to amyloid-related diseases. The aggregation of amyloid fibrils can be associated with pathologies observed in a wide range of diseases known as amyloidoses. For example, amyloid fibrils which aggregate in the brain and central nervous system, are related to Alzheimer’s and Parkinson’s diseases (2). Another example is a prion conversion, where ingested misfolded proteins can seed the aggregation of their homologous polypeptide sequence (3). Similar mechanisms can trigger other amyloidoses (4), but experimental data considering such phenomena are dispersed and often very incompatible.

The importance of interactions between amyloid proteins makes them a subject of numerous experimental studies. However, reviews on interactions between amyloid proteins show that available experimental results are often contradictory (5). Although the information on amyloid proteins is collected in several databases (6–10), until now there has been no database consolidating the results of numerous experiments studying interactions of amyloids. Importantly, existing efforts to present the overview of amyloid–amyloid interactions do not allow for a more in-depth inspection of data (11).

Other problems arise from the lack of clear definitions of field. Although there have been attempts to standardize the vocabulary (12,13) or a list of requirements necessary in reporting amyloid studies (14), the practices are still not being fully implemented. Therefore, comparing different studies, especially those regarding amyloid–amyloid interactions, is problematic and even fundamental concepts may be understood incompatibly.

Therefore, we designed a structured vocabulary to describe amyloid–amyloid interactions more rigorously. It covers descriptors that fully define the exact nature of the influence of an interactor on an interactee. Using the proposed methodology, we manually curated a majority of reported interactions between amyloids and presented this information in the form of an interactive graph and a tabular database.

## MATERIALS AND METHODS

### Standardized terminology

**Figure 1.**
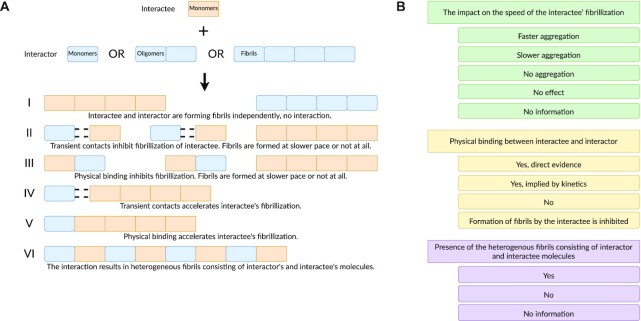
(**A**) Six scenarios of interactions between amyloid proteins. Orange and blue tiles denotes molecules of amyloid-like proteins participating in interactions. Roman numerals denote different interactions scenarios. (**B**) Three descriptors of AmyloGraph. Rectangles represent the descriptors. Rectangles with round edges represent the levels of the descriptors. Green, yellow and purple represent descriptors 1, 2 and 3, respectively.

To describe interactions between amyloid proteins, we created a precisely controlled vocabulary. First, we defined six possible scenarios of amyloid–amyloid interactions (Figure 1A). All scenarios assume that there are only two participants in each interaction, and an interactor modulates self-assembly of an interactee. We are aware that in reality the distinction between the interactee and interactor may not always be clear or, depending on other factors, a specific interaction can fall under more than one scenario at the same time. However, this simplification allowed us to better design the standardized terminology and led to a more structured description of interactions between amyloid proteins.

Next, we developed three descriptors to more rigorously describe details of the scenarios, based on one of the following: the fibrillization speed, presence of physical binding between both interacting proteins and appearance of heterogeneous fibrils (Figure 1 B).

Each descriptor provides specific dictionary terms of possible states. For example, descriptor I, ‘The impact on the speed of fibrillization’, enables the choice of one of the following states: ‘faster fibrillization’, ‘slower fibrillization’, ‘no fibrillization’, ‘no effect’ and ‘no information’. The designed states are mutually exclusive and provide in-depth description to relate them to relevant experimental results (Supplementary Information, section *Descriptors* and Supplementary Figure S1).

It is essential to stress that most publications provided information only on the interactee’s (a protein whose self-assembly is modulated by interactee) ability to create amyloid-like fibrils. Therefore, the descriptors focus only on the behavior of interactee. However, some manuscripts reported on the self-assembly of both interactee and interactor. In this case, such interactions were reported bidirectionally, where protein A acts as an interactor and protein B as the interactee, and *vice versa*.

Our descriptors do not replace existing terminology, but rather standardize it. For example, a combination of answers to descriptor I ‘The impact on the speed of fibrillization’: ‘Faster aggregation’ and descriptor II ‘Physical binding between interactor and interactee’: ‘Yes, direct evidence’ or ‘Yes, implied by kinetics’ can be related to either cross-seeding or co-incubation (12). These two experiments are drastically different design-wise: co-incubation requires both interactee and interactor to be in monomeric form, while in cross-seeding experiment interactee is monomeric and interactor in the form of small aggregates. However, studies frequently do not mention the exact form of interactee and interactor making general descriptors easier to use and more accurate.

### Database scope

The scope of the current AmyloGraph version is limited to interactions between two proteins, each of them able to form an amyloid-like aggregate by its wild type. Additionally, we allowed for non-aggregating homologs of a well-known amyloid protein, such as rat amylin (15).

While selecting the source publications, we focused on *in vitro* studies published after 2000. The complete list of eligibility criteria is available in the Supplementary Information, section *Manuscript collection*.

### Data acquisition and curation

To ensure the highest possible quality of the collected data, the data acquisition and curation were executed in a three-stage pipeline, including: pre-screen of manuscripts, manual curation and independent final validation. Importantly, the first two steps were supported by dedicated forms which played a crucial role in standardizing annotations provided by curators.

The pre-screen of manuscripts started with our in-house collection of 24 publications. Next, we expanded the search by repeatedly adding manuscripts cited by manuscripts or referencing manuscripts from our collections. The final collection included 562 manuscripts, out of which 364 were putatively suitable for the database. Although our collection system was laborious, we found it to be more effective than a search of PubMed records based on its annotations (Supplementary Figure S2A).

Next, the database curators manually extracted information on interactions from the suitable manuscripts. It should be emphasized that, in the curation procedure, we did not re-interpret data and conclusions provided by their authors. Curators interpreted the data only if the authors did not provide a description of the results or if the existing description was too limited. To help curators interpret the results, we enhanced the descriptors with the specifics of experimental procedures, which helped them to identify a descriptor level and draw the best final conclusion. Moreover, during the project, we developed a FAQ list of almost 100 questions related to the curation procedure, which helped the curators. Thanks to all these precautions, the curators could provide data of a higher quality.

During the initial curation, curators reviewed all collected manuscripts. Curators annotated these interactions using our three descriptors and collected information on sequences of proteins participating in the interaction, focusing on the presence of mutations or other alterations. Curators were also obliged to preserve parts of the publication (in the graphical or textual form) supporting their decisions regarding final description in the records.

After the initial curation, we validated all the collected data to further increase their quality. In this procedure, new curators reviewed the assigned interaction records. The semi-random assignment procedure ensured that the curator who validated a specific record was not involved in its initial curation. Finally, the correct records were accepted in the database. The manual curation resulted in 172 publications and 883 interactions (Supplementary Figure S2B).

Next, we contacted the authors of all 172 publications included in AmyloGraph to obtain their validation of our records. We always tried to reach the corresponding authors or, in case of their unavailability, the publication’s first author. The authors were provided with customized links to Google Sheets only containing data from manuscripts they authored. In total, we contacted 122 authors, and 11 authors (9.04%) confirmed 81 interactions (9.17%) in 21 manuscripts (12.14%). Despite our efforts, we could not find correct and up-to-date contact information to authors of three manuscripts. It is important to notice that no interactions were removed or added after the contact with the authors, which implies a high data quality (Supplementary Figure S2C).

The in-depth description of the curation procedure is available in the Supplementary Information, section *Data acquisition*. The full list of 172 manuscripts is available in the Supplementary Information, section *Supplementary references*.

### Implementation

One of the main limitations of web-based tools is their in-built reliance on the external servers which reduces their persistence (16). Therefore, we made AmyloGraph fully deployable and usable even if the main server is no longer available. To do so, we implemented our tool as an R package (17). The package contains also the front-end of our database, available as a Shiny app (18). The local deployment of AmyloGraph only requires a very rudimentary knowledge of R and is described in the AmyloGraph main repository at https://kotulskalab.github.io/AmyloGraph/. The AmyloGraph codebase is open and documented in the roxygen2 standard.

## DATABASE OVERVIEW

**Figure 2.**
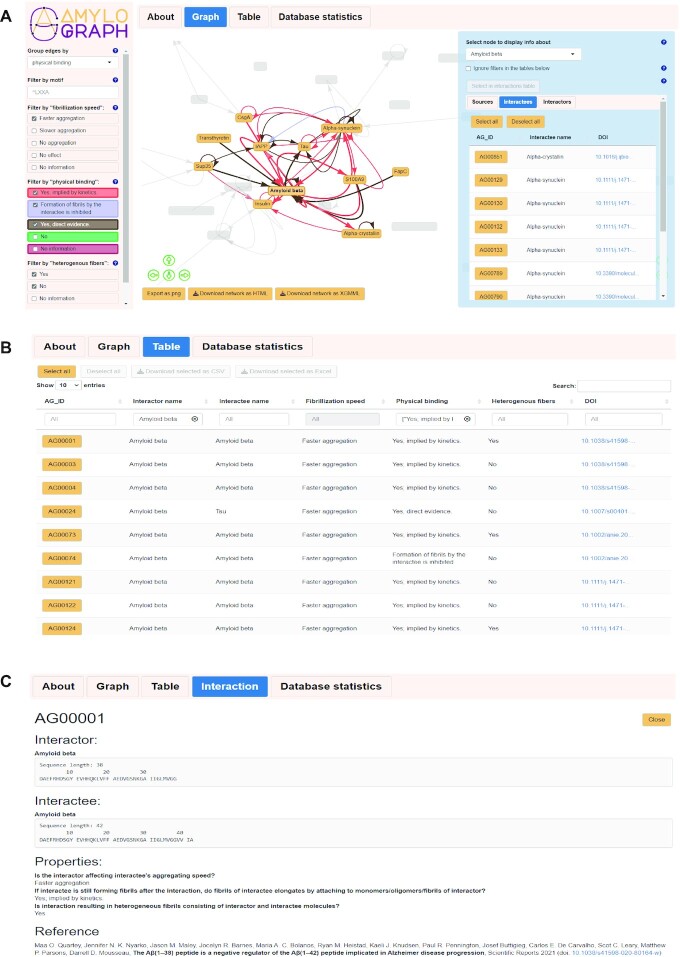
Overview of the AmyloGraph database. (**A**) Graph view of interactions between amyloid proteins. The interactions (edges of the graph) are colored according to the levels of descriptor 2, ‘physical binding’. The panel on the right-hand side represents an overview of the amyloid-β interactions. (**B**) Tabular view of interactions. The top section of this card contains download options allowing to obtain data in a selected flat-file format. (**C**) View of a single interaction with the sequential information.

Manually curated data, obtained in the previously described procedure, are available in the AmyloGraph database. Currently, the database includes 883 interactions between 46 proteins reported in 172 manuscripts. Furthermore, one of the main objectives of the database is to present the interactions between amyloid proteins in a standardized manner and user-friendly presentations, such as a graph format (Figure 2A). Here, nodes represent individual amyloid proteins and edges stand for interactions between them. Notably, a single edge represents all interactions between two amyloid proteins. Tooltips of the edges represent digital object identifiers (DOIs) of manuscripts reporting the interactions.

After clicking on a node, a panel on the right-hand side opens. It presents brief information on a protein, its name and links to its UniProt record. If a single amyloid protein in AmyloGraph is associated with several records in the UniProt, we provide links to all of them. This panel also contains two tables presenting all interactees and interactors of the protein.

Aside from the graph, AmyloGraph enables tabular representation of the interaction data (Figure 2B). The table is interactive and searchable. A user can also download selected rows in a flat-table format (.csv or .xlsx). As a result, the downloaded table contains all available information, including the sequences of amyloid proteins participating in the interactions.

Both, graph and tabular representations of the data can be filtered out using filters available on the left-hand side of the user interface. The filters cover all three descriptors. Moreover, a user can color the edges on the graph, according to the levels of a chosen descriptor. The user can also use amino acid sequences to filter the information presented in the graph or tabular form. Here, we implemented a simplified set of regular expression inspired by the POSIX system to facilitate more advanced searches.

The last card of the graphical interface, ‘Interaction’, opens when the user selects a specific interaction (Figure 2C). This view presents information on a single interaction, including levels of all descriptors and exact sequences of proteins. In case of multi-chain proteins, such as insulin, AmyloGraph presents the sequences of all chains.

To streamline the use of AmyloGraph, we enhanced it with helpers explaining basic functionalities of the database. Moreover, a video tutorial is available, presenting examples of AmyloGraph queries.

AmyloGraph is a FAIR-compliant database (19). All interactions are identifiable by an individual index. They are also linked to original publications using their DOIs provided by the Crossref. Proteins participating in interactions are linked to the UniProt database (20). As recommended by the FAIR guidelines, we extended the existing vocabulary to describe our data by fully providing our standardized methodology.

## CONCLUSIONS AND FUTURE DIRECTIONS

AmyloGraph is the first endeavor to present an overview of experimentally verified interactions between amyloid proteins. It has also been the first attempt to standardize reporting of the amyloid–amyloid interactions and present them in the interactive database. We believe that, thanks to our rigorous data curation procedure, we have managed to collect and thoroughly systematize the majority of available information. Even though AmyloGraph is currently the most comprehensive compendium on the interactions between amyloid proteins, we see three areas that require an improvement: constant updates, representation of protein data and extending information of experimental conditions.

The greatest challenge regarding AmyloGraph, which we envisage, will be to keep it updated. We encountered a sudden influx of new publications reporting new interactions during our work on the database. To alleviate this issue, AmyloGraph offers a submission form for authors involved in relevant research to report their results directly to the database. Thus, we are going to implement a highly structured system for finding publications by annotating records acquired in searches of the PubMed database.

One of other challenges regarding AmyloGraph is representation of protein data. Right now, AmyloGraph is very protein-centric and treats whole families of homologs or variants of a single protein as a single entity. The actual situation is much more complicated as we often deal with fragments of recombinant proteins or even protein grafts (21). In the future, we want to extend AmyloGraph to contain more information about the protein sequence and allow proteins with non-standard amino acids or even non-amino-acid modifications. This change is also necessary to add to AmyloGraph information on the impact of small molecules on amyloid fibrillization.

Another limitation of AmyloGraph, which we plan to alleviate in the next version, is the lack of experimental information. Amyloid assembly process and their interactions are extremely liable to experimental conditions, such as pH (22) or concentration of proteins (23). We are convinced that each record in AmyloGraph should be annotated with more parameters defining the environment of the interaction.

Even considering all limitations described above, we still believe that the current release of AmyloGraph is a valuable tool that provides access to a unique dataset. To our knowledge, AmyloGraph is the first effort to collect and present information on interactions between amyloid proteins in a unified format. AmyloGraph’s high accessibility and data quality further enhance its usefulness.

## DATA AVAILABILITY

The forms supporting collection of manuscripts, initial curation and validation of manuscripts are available upon request to the corresponding author. The procedure of data acquisition, the in-depth definitions of AmyloGraph descriptors and references to all curated manuscripts, are available in the Supplementary Information and online at https://kotulskalab.github.io/AmyloGraph/articles/definitions.html. AmyloGraph is available as an online database (http://AmyloGraph.com/).

## CODE AVAILABILITY

All AmyloGraph functionalities are also accessible as the R package (https://github.com/KotulskaLab/AmyloGraph).

## Supplementary Material

gkac882_Supplemental_File
