# Supplementary material for: AmyloGraph: a comprehensive database of amyloid–amyloid interactions
Source: Nucleic Acids Res. 2022 Oct 16;51(D1):D352–7. doi: 10.1093/nar/gkac882 (PMC9825533; doi:10.1093/nar/gkac882)
Supplement: gkac882_Supplemental_File [file gkac882_supplemental_file.pdf]

AmyloGraph: supplementary materials

Michał Burdukiewicz Dominik Rafacz Agnieszka Barbach
Katarzyna Hubicka Laura Bąkała Anna Lassota Jakub Stecko
Natalia Szymańska Jakub W. Wojciechowski Dominika Kozakiewicz
Natalia Szulc Jarosław Chilimoniuk Izabela Jęśkowiak
Marlena Gąsior-Głogowska Małgorzata Kotulska

Contents

General assumptions **1**
Main definitions . . . 1
General interaction scenarios . . . 2
Descriptors: . . . 2
Descriptor 1. The impact on the speed of the interactee' fibrillization. . . . 2
Descriptor 2. Physical binding between interactee and interactor. . . . 3
Descriptor 3. Presence of the heterogenous fibrils consisting of interactor and interactee molecules. . . . 3
Information on the sequence of interactor and interactee . . . 4

Data acquisition **4**
Manuscript collection . . . 4
Initial curation . . . 5
Validation . . . 5
Contact with authors . . . 5

Usage of AmyloGraph **6**
Filter by motif . . . 6

Supplementary references **6**

General assumptions

Main definitions

1. An **interactee** is always acted upon by an **interactor**. This is the AmyloGraph terminology and it is not used in analyzed manuscripts. Suppose the publication uses words like 'co-incubation' and from the text itself it is not clear how to distinguish between interactor and interactor. Additionally, the authors study the effect of Protein A on Protein B and vice versa. In this case, we annotate it as two separate interactions: I) A is an interactor over B and II) B is an interactor over A.
2. General logic. We use following operators AND and OR which are defined in the following way:
   1. Operator OR applied to a and b means: I) a, II) b, III) a and b.
   2. Operator AND applied to a and b only when we have a and b simultaneously.
3. Authors' interpretation always supersedes ours.
4. We refrain from using vague terms as seeding or cross-seeding. Instead, we describe the interaction using three descriptors (described in the section Descriptors).

5. We refer to glossary provided by MIRRAGGE – Minimum Information Required for Reproducible AGGregation Experiments (doi: 10.3389/fnmol.2020.582488).
    1. Additional terms:
        - **T50**: the time required for the amyloid reaction to reach 50% of the final fluorescence intensity.

General interaction scenarios

We assume that six main scenarios can occur during the interaction of two amyloid proteins (see the figure above). Scenarios depend on the stability (permanent/transient) of the binding between interactor and interactee and the impact on the interactee's fibrillization speed (acceleration/inhibition). If there is no interaction between interactor and interactee, amyloid proteins are forming fibrils independently - scenario I occurs. If there is transient contact between interactor and interactee along with fibrillation inhibition - scenario II takes place, but if interactee's fibrillation is accelerated - scenario IV. If the physical binding between interactor and interactee occurs together with fibrillation inhibition - scenario III happens, but if interactee's fibrillation is accelerated - scenario V or VI takes place.

scenario I: no interaction between interactor and interactee & amyloid proteins are forming fibrils independently scenario II: transient contact between interactor and interactee & inhibition of homofibril fibrillization scenario III: physical binding between interactor and interactee & inhibition of homofibril fibrillization scenario IV: transient contact between interactor and interactee & acceleration of homofibril fibrillization scenario V: physical binding between interactor and interactee & acceleration of homofibril fibrillization scenario VI: physical binding between interactor and interactee & acceleration of heterofibril fibrillization

The scenarios are discrete but they represent points in the continuum rather than the real phenomenons. We are aware that depending on the experimental conditions an interaction can vary between scenario III and IV. Therefore, we do not imply that each interaction follows strictly one of these scenarios, but rather presents most dominantly one of them. To distinguish between these interaction scenarios we design three descriptors (described below). Descriptor 1. differentiates between scenarios I (no effect on kinetics) II and III (inhibited aggregation) as well as IV, V and VI (acceleration). Descriptor 2. discriminates between scenarios IV and V/VI. Descriptor 3. differentiates between scenarios V and VI.

For example, if descriptor 1 is **faster aggregation**, descriptor 2 - **yes, direct evidence** and descriptor 3 - **yes**, **no** or **no information**, they describe cross-seeding.

Descriptors:

Descriptor 1. The impact on the speed of the interactee' fibrillization.

General remarks: this descriptor is fully based on the kinetics or any kinetic data. Here, by fibrillization we mean aggregation from low-organisation levels to mature fibrils confirmed by e.g., microscopy images. If the interactor accelerates the speed of the oligomer formation, but they never aggregate into the level of mature fibrils (fibrillization does not occur), it is not an acceleration as we understand it. The commonly used technique to measure the kinetics of fibrillization is Thioflavin T (ThT) assay (e.g., ThT 101: a primer on the use of thioflavin T to investigate amyloid formation (doi: 10.1080/13506129.2017.1304905)). We are aware of the fact ThT is not always quantitative, i.e. a higher (or lower) ThT level - under different conditions (e.g., the presence of the interactor) - can be caused by changes to the fibril structure rather than the amount of fibrils. For the purpose of simplification, we ignore it and always follow the interpretation of authors.

1. **Faster aggregation**: a) the maximum ThT emission observed at the end of the reaction of the interactee and interactor is higher than maximum ThT emission for interactee alone OR b) if the slope of the kinetic curve is steeper OR c) the lag phase is shorter OR d) T50 is lower. The fibrillization still occurred.
2. **Slower aggregation**: a) the maximum ThT emission observed at the end of the reaction of the interactee and interactor is lower than maximum ThT emission for interactee alone AND b) the slope

of the kinetic curve is less steep) OR c) the lag phase is longer. So we need (a AND b) OR c. The fibrillization still occurred.

3. **No aggregation**: there is no confirmed fibrilization after the interaction.
4. **No effect**: a) The slopes of kinetic curves are visibly similar AND b) the maximum ThT emission is similar AND c) the lag phase is similar.
5. **No information**: there were no kinetic assays.

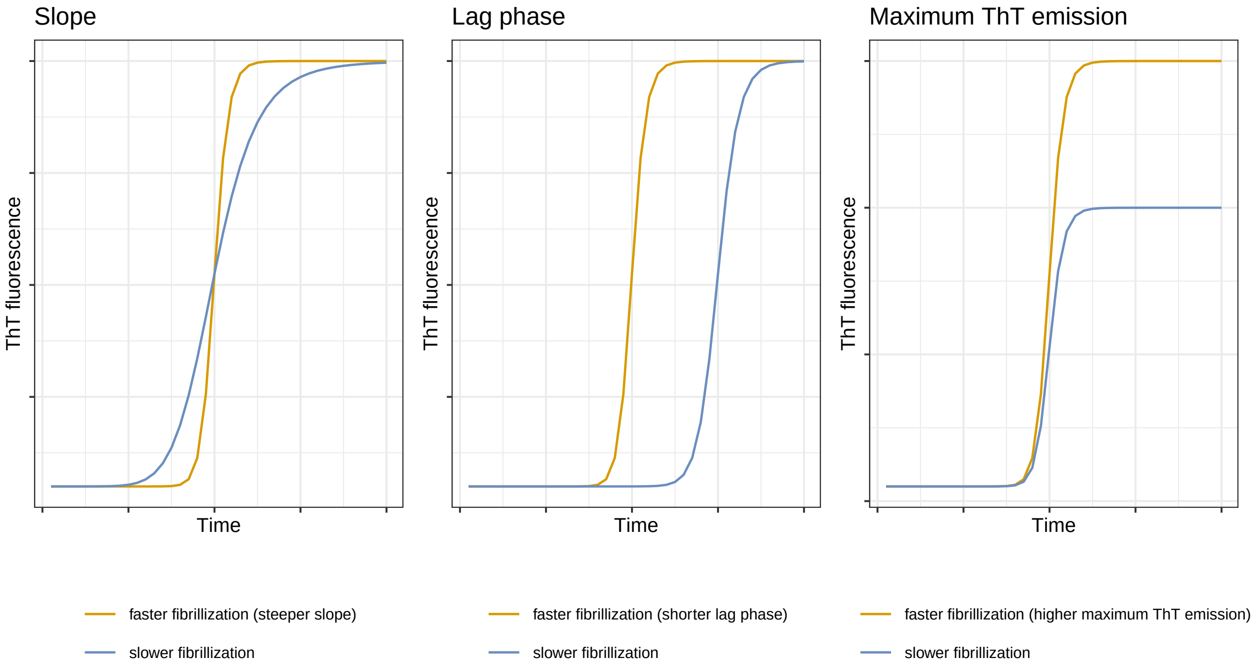

Figure S1: Comparison of ThT curves.

Descriptor 2. Physical binding between interactee and interactor.

1. **Yes, direct evidence**: there is an experimental evidence that fibrils consist of two different amyloids (labeling; immunolabeling). It also applies if we have a visible colocalization of an interactee and an interactor visible in the microscopic images.
2. **Yes, implied by kinetics**: if seeding is implied by kinetic experiments results and as such it is interpreted by authors of the publication. In principle, this answer covers every acceleration of the fibrillization confirmed by kinetic experiments.
3. **No**: no effect on the elongation of interactee's fibrils.
4. **Formation of fibrils by the interactee is inhibited**: the formation of interactee's aggregates was slowed or completely halted by the interactor.
5. **No information**: there is no experimental evidence and seeding is not implied by kinetics experiments results.

Descriptor 3. Presence of the heterogenous fibrils consisting of interactor and interactee molecules.

1. **Yes:** applies when a) there is experimental evidence that fibrils consist of two different amyloids (labeling; immunolabeling) AND b) the mature fibrils are structurally different than fibrils formed in the presence of interactor OR c) the term co-aggregation/ heterogeneous fibrils/ hybrid fibrils is used to describe the aggregation process.
2. **No:** if the resulting amyloid fibrils have the dimension matching that of the aggregating interactee alone. a) the mature fibrils are confirmed by a microscopy technique to have the same structure as

fibrils formed by the interactee without the presence of the interactor OR b) there is no fibrillar product at all OR c) an interactee and an interactor are the same protein.
3. **No information**: there is no experimental evidence and seeding is not implied by kinetics experiments results.

Information on the sequence of interactor and interactee

Name of the amyloid protein: was chosen from a list of amyloid proteins considered by us. Every protein on the list has confirmed amyloid-like properties.

Sequence: The sequence is a vector of amino acids.

1. In the case when the exact sequence is not known, we provide the longest possible precursor from UniProt.
2. If protein is available only in a complex or was isolated (for example, purified from isolated spleen amyloid fibrils), we consider sequence as unavailable.
3. If interactee or interactor are mutants/fragments of an amyloid protein, we provide only the sequence of the mutant/fragment and not the wild type protein.
4. We consider sequences that have modified amino acids (e.g., methylated), but we do not include this information in the sequence.
5. We do NOT consider mutants that instead of standard amino acids have a) non-biogenic amino acids (e.g., tyramine) b) non-amino acid linkers.
6. If the sequence of the interactor or the interactee contains modified amino acid residues (e.g., phospory-lated), we do not supply this information in the sequential data.

Source sequence: the UniProt ID of the original protein.

The AmyloGraph database as a single protein treats a protein that can occur in many taxonomic variants or after modifications (e.g., we have human and bovine precursor albumins, P02768 and P02769 as well as the products of the post-translational modifications, Q56G89).

The source sequence may be not identical to the interactor's or interactee's sequence. However, interactor or interactee might be a part of the source sequence (as human amyloid beta 1-40 is a part of the P05067) or a mutated variant of a source sequence (when some amino acids are altered compared to the original sequence). For example, in AmyloGraph database the CsgA protein can occur as one of 6 variants, including 4 homologues and 2 mutants.

Data acquisition

Manuscript collection

We started our manuscript collection on amyloid-amyloid interactions by defining the eligibility criteria:

1. The manuscript has to be published after 2000.
2. The manuscript has to report directly experimental results (this excludes review papers and simula-tions).
3. The manuscript has to report experiments conducted *in vitro*.
4. The manuscript has to report interactions leading to fibrillization.
    1. If the interactor accelerates the speed of the oligomer formation, but they never aggregate into the level of mature fibrils (fibrillization does not occur), it is not an acceleration in our understanding, but inhibition.
    2. In the case of different interactions of the same two amyloids, when these differences stem from the different amyloid formation levels (monomer, oligomer, fiber), pH, concentration, tempera-ture or other experimental conditions, we showcase these interactions as two (or more) different interactions.
5. The manuscript has to report interactions between two amyloid proteins. The list of amyloid proteins considered by us is available here.

   1. If one of the interaction participants is a non-amyloid protein, it should not be included in the database. The only exceptions are: a) non-aggregating homologs of known amyloid proteins b) non-aggregating mutants of amyloid proteins c) non-aggregating fragments of amyloid proteins.
   2. If the interactee or interactor is a) a mutant of an amyloid protein OR b) a fragment of an amyloid protein OR c) a taxonomic variant of an amyloid protein, we still add them to the database under the name of the original protein. However, in this case, we provide the exact sequence of the interactee/interactor and not the original protein.
   3. If the sequence of the interactor or the interactee contains (due to modifications) non-amino acids or nonbiogenic amino acids, this interaction is rejected.
6. The manuscript has to report only two-party interactions. The database does not contain interactions with more than two participants, and the only exception is when two out of three participants are the same protein in a different aggregation level.

We have started our search with the analysis of 24 manuscripts in our in-house collection of publications. Next, we have expanded our search by repeatedly adding manuscripts cited by manuscripts in our collection or referencing manuscripts in our collections. The final collection had 364 manuscripts.

We have curated the information in collected publications using a two-step procedure: **initial curation** and **validation**.

Initial curation

During this procedure, a curator reviewed all interactions described in the manuscripts and annotated them in the dedicated form considering three AmyloGraph descriptors: descriptor 1. the impact on the speed of the fibrillization; descriptor 2. physical binding between interactee and interactors; descriptor 3. presence of the heterogenous fibrils (described in detail in the section Descriptors). They chose names of amyloid proteins involved in the interaction from a list and collected information on the amyloids' sequence. Each record was associated with manuscript's doi.

The final list of interactions after the initial curation covered 863 interactions 49 proteins described in 185 manuscripts.

Validation

During this procedure, a curator has independently reviewed the reported interaction records from assigned manuscripts in the dedicated form. The semi-random assignment procedure ensured that the curator who validated a specific record was not involved in its initial curation.

They reviewed interaction records similarly to during the initial curation step. A curator considered three AmyloGraph descriptors: descriptor 1. the impact on the speed of the fibrillization; descriptor 2. physical binding between interactee and interactors; descriptor 3. presence of the heterogenous fibrils Descriptors. They chose amyloid proteins' names from a list, collected information on the sequence of amyloid proteins involved in the interaction, and provided the sequence of an original protein by its UniProt ID. They could also add in missing interaction records or remove false ones.

The final list covers 883 interactions between 46 proteins described in 172 manuscripts.

Contact with authors

We consulted the final result of the validation with the authors of manuscripts reporting given interactions. To do so, we contacted corresponding author. In the case of more than two corresponding authors, we took the very last author of the publication. If the corresponding author was not available, we tried to contact the first authors' of the publication. If somebody authored more then one manuscript we contacted this author about all of the reported interactions.

We contacted 122 authors. 11 authors confirmed 81 interactions (9.17 %) in 21 manuscripts (12.21 %). Despite our efforts, we could not find a way to contact the authors of three manuscripts.

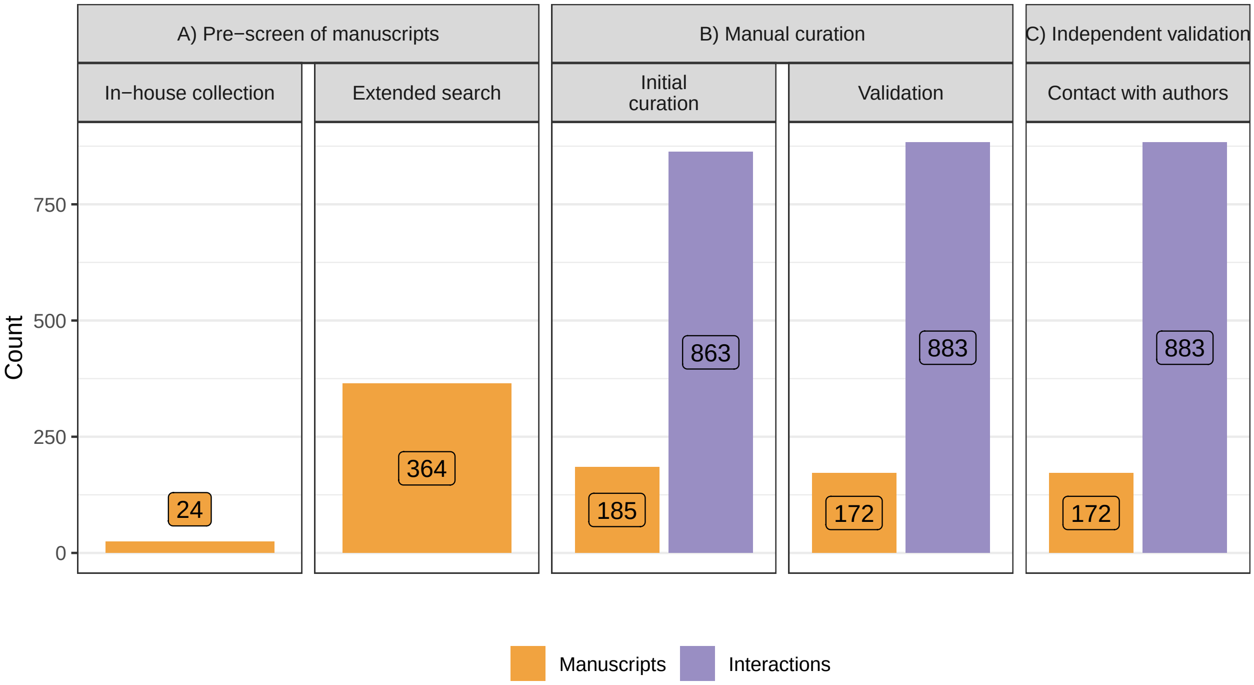

Figure S2: Number of manuscripts, proteins and interactions during the different stages of data curation.

Usage of AmyloGraph

Filter by motif

Data in AmyloGraph can be filtered using an amino acid motif. A motif that should appear in either interactor's or interactee's sequence. Only interactions between those sequences will be displayed on the graph and in the table.

A motif should consist of the letters representing amino acids with possibility of using the following ambiguous letters: * "B" – either "D" or "N" * "J" – either "I" or "L" * "Z" – either "E" or "Q" * "X" – any standard amino acid

Additionally, the character "*" may be used for a subsequence of any (possibly distinct) amino acids of any length. The character "^" may be used as the first character of a motif to mark the beginning of the sequence. Similarly, "$" may be used as the last character of a motif to mark the end of a sequence.

Some exemplary motifs:

- "A" – any sequence containing "A"
- "GLAAALGA" – any sequence containing "GLAAALGA"
- "^AAAAA" – any sequence starting with "AAAAA"
- "AXXXA" – any sequence containing two "A"s with exactly three amino acids between them
- "A*A" – any sequence containing two "A"s and any number of any amino acids between them
- "^A*GG$" – any sequence starting with a single "A" and ending with a double "G"
- "^AG$" – a sequence that is exactly "AG"

Supplementary references

The articles listed below are the sources of curated data in the AmyloGraph database.

1. Emil Dandanell Agerschou, Marie P. Schützmann, Nikolas Reppert, Michael M. Wördehoff, Hamed

Shaykhalishahi, Alexander K. Buell, Wolfgang Hoyer, **-Turn exchanges in the -synuclein segment 44-TKEG-47 reveal high sequence fidelity requirements of amyloid fibril elongation**, Biophysical Chemistry 2021 (doi: 10.1016/j.bpc.2020.106519).

2. Mohsen Akbarian, Maryam Kianpour, Reza Yousefi, Ali Akbar Moosavi-Movahedi, **Characterization of insulin cross-seeding: the underlying mechanism reveals seeding and denaturant-induced insulin fibrillation proceeds through structurally similar intermediates**, RSC Advances 2020 (doi: 10.1039/d0ra05414c).

3. Erika Andreetto, Li-Mei Yan, Andrea Caporale, Aphrodite Kapurniotu, **Dissecting the Role of Single Regions of an IAPP Mimic and IAPP in Inhibition of A 40 Amyloid Formation and Cytotoxicity**, ChemBioChem 2011 (doi: 10.1002/cbic.201100192).

4. Erika Andreetto, Eleni Malideli, Li-Mei Yan, Michael Kracklauer, Karine Farbiarz, Marianna Tatarek-Nossol, Gerhard Rammes, Elke Prade, Tatjana Neumüller, Andrea Caporale, Anna Spanopoulou, Maria Bakou, Bernd Reif, Aphrodite Kapurniotu, **A Hot-Segment-Based Approach for the Design of Cross-Amyloid Interaction Surface Mimics as Inhibitors of Amyloid Self-Assembly**, Angewandte Chemie International Edition 2015 (doi: 10.1002/anie.201504973).

5. Shruti Arya, Sarah L. Claud, Kristi Lazar Cantrell, Michael T. Bowers, **Catalytic Prion-Like Cross-Talk between a Key Alzheimer's Disease Tau-Fragment R3 and the Type 2 Diabetes Peptide IAPP**, ACS Chemical Neuroscience 2019 (doi: 10.1021/acschemneuro.9b00516).

6. Joseph D. Barritt, Nadine D. Younan, John H. Viles, **N-Terminally Truncated Amyloid- (11 – 40/42) Cofibrillizes with its Full-Length Counterpart: Implications for Alzheimer's Disease**, Angewandte Chemie International Edition 2017 (doi: 10.1002/anie.201704618).

7. Karishma Bhasne, Sanjana Sebastian, Neha Jain, Samrat Mukhopadhyay, **Synergistic Amyloid Switch Triggered by Early Heterotypic Oligomerization of Intrinsically Disordered -Synuclein and Tau**, Journal of Molecular Biology 2018 (doi: 10.1016/j.jmb.2018.04.020).

8. Henrik Biverstål, Lisa Dolfe, Erik Hermansson, Axel Leppert, Mara Reifenrath, Bengt Winblad, Jenny Presto, Jan Johansson, **Dissociation of a BRICHOS trimer into monomers leads to increased inhibitory effect on A 42 fibril formation**, Biochimica et Biophysica Acta (BBA) - Proteins and Proteomics 2015 (doi: 10.1016/j.bbapap.2015.04.005).

9. David C. Bode, Helen F. Stanyon, Trisha Hirani, Mark D. Baker, Jon Nield, John H. Viles, **Serum Albumin's Protective Inhibition of Amyloid- Fiber Formation Is Suppressed by Cholesterol, Fatty Acids and Warfarin**, Journal of Molecular Biology 2018 (doi: 10.1016/j.jmb.2018.01.008).

10. Erin Bove-Fenderson, Ryo Urano, John E. Straub, David A. Harris, **Cellular prion protein targets amyloid- fibril ends via its C-terminal domain to prevent elongation**, Journal of Biological Chemistry 2017 (doi: 10.1074/jbc.m117.789990).

11. Kristoffer Brännström, Tohidul Islam, Anna L. Gharibyan, Irina Iakovleva, Lina Nilsson, Cheng Choo Lee, Linda Sandblad, Annelie Pamrén, Anders Olofsson, **The Properties of Amyloid- Fibrils Are Determined by their Path of Formation**, Journal of Molecular Biology 2018 (doi: 10.1016/j.jmb.2018.05.001).

12. Samuel J. Bunce, Yiming Wang, Katie L. Stewart, Alison E. Ashcroft, Sheena E. Radford, Carol K. Hall, Andrew J. Wilson, **Molecular insights into the surface-catalyzed secondary nucleation of amyloid- 40 (A 40) by the peptide fragment A 16–22**, Science Advances 2019 (doi: 10.1126/sciadv.aav8216).

13. Jason Candreva, Edward Chau, Margaret E. Rice, Jin Ryoun Kim, **Interactions between Soluble Species of -Amyloid and -Synuclein Promote Oligomerization while Inhibiting Fibrillization**, Biochemistry 2019 (doi: 10.1021/acs.biochem.9b00655).

14. Ping Cao, Fanling Meng, Andisheh Abedini, Daniel P. Raleigh, **The Ability of Rodent Islet Amyloid Polypeptide To Inhibit Amyloid Formation by Human Islet Amyloid Polypeptide**

Has Important Implications for the Mechanism of Amyloid Formation and the Design of Inhibitors, Biochemistry 2010 (doi: 10.1021/bi901751b).

15. Linda Cerofolini, Enrico Ravera, Sara Bologna, Thomas Wiglenda, Annett Böddrich, Bettina Purfürst, Iryna Benilova, Magdalena Korsak, Gianluca Gallo, Domenico Rizzo, Leonardo Gonnelli, Marco Fragai, Bart De Strooper, Erich E. Wanker, Claudio Luchinat, **Mixing A (1–40) and A (1–42) peptides generates unique amyloid fibrils**, Chemical Communications 2020 (doi: 10.1039/d0cc02463e).

16. Yu-Jen Chang, Yun-Ru Chen, **The coexistence of an equal amount of Alzheimer's amyloid-40 and 42 forms structurally stable and toxic oligomers through a distinct pathway**, FEBS Journal 2014 (doi: 10.1111/febs.12813).

17. Saketh Chemuru, Ravindra Kodali, Ronald Wetzel, **C-Terminal Threonine Reduces A 43 Amyloidogenicity Compared with A 42**, Journal of Molecular Biology 2016 (doi: 10.1016/j.jmb.2015.06.008).

18. Sean Chia, Patrick Flagmeier, Johnny Habchi, Veronica Lattanzi, Sara Linse, Christopher M. Dobson, Tuomas P. J. Knowles, Michele Vendruscolo, **Monomeric and fibrillar -synuclein exert opposite effects on the catalytic cycle that promotes the proliferation of A 42 aggregates**, Proceedings of the National Academy of Sciences 2017 (doi: 10.1073/pnas.1700239114).

19. Line Friis Bakmann Christensen, Kirstine Friis Jensen, Janni Nielsen, Brian Stougaard Vad, Gunna Christiansen, Daniel Erik Otzen, **Reducing the Amyloidogenicity of Functional Amyloid Protein FapC Increases Its Ability To Inhibit -Synuclein Fibrillation**, ACS Omega 2019 (doi: 10.1021/acsomega.8b03590).

20. R. Costa, A. Gonçalves, M.J. Saraiva, I. Cardoso, **Transthyretin binding to A-Beta peptide - Impact on A-Beta fibrillogenesis and toxicity**, FEBS Letters 2008 (doi: 10.1016/j.febslet.2008.02.034).

21. Ellen Y. Cotrina, Ana Gimeno, Jordi Llop, Jesús Jiménez-Barbero, Jordi Quintana, Gregorio Valencia, Isabel Cardoso, Rafel Prohens, Gemma Arsequell, **Calorimetric Studies of Binary and Ternary Molecular Interactions between Transthyretin, A Peptides, and Small-Molecule Chaperones toward an Alternative Strategy for Alzheimer's Disease Drug Discovery**, Journal of Medicinal Chemistry 2020 (doi: 10.1021/acs.jmedchem.9b01970).

22. Risto Cukalevski, Xiaoting Yang, Georg Meisl, Ulrich Weininger, Katja Bernfur, Birgitta Frohm, Tuomas P. J. Knowles, Sara Linse, **The A 40 and A 42 peptides self-assemble into separate homomolecular fibrils in binary mixtures but cross-react during primary nucleation**, Chemical Science 2015 (doi: 10.1039/c4sc02517b).

23. Luisa D'Urso, Marcello Condorelli, Orazio Puglisi, Carmelo Tempra, Fabio Lolicato, Giuseppe Compagnini, Carmelo La Rosa, **Detection and characterization at nM concentration of oligomers formed by hIAPP, A (1–40) and their equimolar mixture using SERS and MD simulations**, Physical Chemistry Chemical Physics 2018 (doi: 10.1039/c7cp08552d).

24. C. Dammers, M. Schwarten, A. K. Buell, D. Willbold, **Pyroglutamate-modified A (3-42) affects aggregation kinetics of A (1-42) by accelerating primary and secondary pathways**, Chemical Science 2017 (doi: 10.1039/c6sc04797a).

25. Anvesh K. R. Dasari, Rakez Kayed, Sungsool Wi, Kwang Hun Lim, **Tau Interacts with the C-Terminal Region of -Synuclein, Promoting Formation of Toxic Aggregates with Distinct Molecular Conformations**, Biochemistry 2019 (doi: 10.1021/acs.biochem.9b00215).

26. Dexter N. Dean, Jennifer C. Lee, **Defining an amyloid link Between Parkinson's disease and melanoma**, Proceedings of the National Academy of Sciences 2020 (doi: 10.1073/pnas.2009702117).

27. Francis C. Dehle, Heath Ecroyd, Ian F. Musgrave, John A. Carver, **B-Crystallin inhibits the cell toxicity associated with amyloid fibril formation by -casein and the amyloid- peptide**, Cell Stress and Chaperones 2010 (doi: 10.1007/s12192-010-0212-z).

28. Irina L. Derkatch, Susan M. Uptain, Tiago F. Outeiro, Rajaraman Krishnan, Susan L. Lindquist, Susan W. Liebman, **Effects of Q/N-rich, polyQ, and non-polyQ amyloids on the de novo formation of the [PSI +] prion in yeast and aggregation of Sup35 in vitro**, Proceedings of the National Academy of Sciences 2004 (doi: 10.1073/pnas.0404968101).

29. Glyn L. Devlin, Tuomas P.J. Knowles, Adam Squires, Margaret G. McCammon, Sally L. Gras, Melanie R. Nilsson, Carol V. Robinson, Christopher M. Dobson, Cait E. MacPhee, **The Component Polypeptide Chains of Bovine Insulin Nucleate or Inhibit Aggregation of the Parent Protein in a Conformation-dependent Manner**, Journal of Molecular Biology 2006 (doi: 10.1016/j.jmb.2006.05.007).

30. Shailendra Dhakal, Courtney E. Wyant, Hannah E. George, Sarah E. Morgan, Vijayaraghavan Rangachari, **Prion-like C-Terminal Domain of TDP-43 and -Synuclein Interact Synergistically to Generate Neurotoxic Hybrid Fibrils**, Journal of Molecular Biology 2021 (doi: 10.1016/j.jmb.2021.166953).

31. Zhi Du, Yijia Guan, Chao Ding, Nan Gao, Jinsong Ren, Xiaogang Qu, **Cross-fibrillation of insulin and amyloid on chiral surfaces: Chirality affects aggregation kinetics and cytotoxicity**, Nano Research 2018 (doi: 10.1007/s12274-018-1995-y).

32. Jiali Du, Regina M. Murphy, **Characterization of the Interaction of -Amyloid with Transthyretin Monomers and Tetramers**, Biochemistry 2010 (doi: 10.1021/bi101280t).

33. Kriti Dubey, Bibin G. Anand, Mayur K. Temgire, Karunakar Kar, **Evidence of Rapid Coaggregation of Globular Proteins during Amyloid Formation**, Biochemistry 2014 (doi: 10.1021/bi501333q).

34. Brian R. Fluharty, Emiliano Biasini, Matteo Stravalaci, Alessandra Sclip, Luisa Diomede, Claudia Balducci, Pietro La Vitola, Massimo Messa, Laura Colombo, Gianluigi Forloni, Tiziana Borsello, Marco Gobbi, David A. Harris, **An N-terminal Fragment of the Prion Protein Binds to Amyloid-Oligomers and Inhibits Their Neurotoxicity in Vivo**, Journal of Biological Chemistry 2013 (doi: 10.1074/jbc.m112.423954).

35. Kanchan Garai, Ammon E. Posey, Xinyi Li, Joel N. Buxbaum, Rohit V. Pappu, **Inhibition of amyloid beta fibril formation by monomeric human transthyretin**, Protein Science 2018 (doi: 10.1002/pro.3396).

36. Ricardo Gaspar, Georg Meisl, Alexander K. Buell, Laurence Young, Clemens F. Kaminski, Tuomas P. J. Knowles, Emma Sparr, Sara Linse, **Secondary nucleation of monomers on fibril surface dominates -synuclein aggregation and provides autocatalytic amyloid amplification**, Quarterly Reviews of Biophysics 2017 (doi: 10.1017/s0033583516000172).

37. Ricardo Gaspar, Tommy Garting, Anna Stradner, **Eye lens crystallin proteins inhibit the autocatalytic amyloid amplification nature of mature -synuclein fibrils**, PLOS ONE 2020 (doi: 10.1371/journal.pone.0235198).

38. Xinwei Ge, Ye Yang, Yunxiang Sun, Weiguo Cao, Feng Ding, **Islet Amyloid Polypeptide Promotes Amyloid-Beta Aggregation by Binding-Induced Helix-Unfolding of the Amyloidogenic Core**, ACS Chemical Neuroscience 2018 (doi: 10.1021/acschemneuro.7b00396).

39. Megan Murray Gessel, Chun Wu, Huiyuan Li, Gal Bitan, Joan-Emma Shea, Michael T. Bowers, **A (39–42) Modulates A Oligomerization but Not Fibril Formation**, Biochemistry 2011 (doi: 10.1021/bi201520b).

40. Seyyed Abolghasem Ghadami, Sean Chia, Francesco Simone Ruggeri, Georg Meisl, Francesco Bemporad, Johnny Habchi, Roberta Cascella, Christopher M. Dobson, Michele Vendruscolo, Tuomas P. J. Knowles, Fabrizio Chiti, **Transthyretin Inhibits Primary and Secondary Nucleations of Amyloid- Peptide Aggregation and Reduces the Toxicity of Its Oligomers**, Biomacromolecules 2020 (doi: 10.1021/acs.biomac.9b01475).

41. Benoit I. Giasson, Mark S. Forman, Makoto Higuchi, Lawrence I. Golbe, Charles L. Graves, Paul T. Kotzbauer, John Q. Trojanowski, Virginia M.-Y. Lee, **Initiation and Synergistic Fibrillization of Tau and Alpha-Synuclein**, Science 2003 (doi: 10.1126/science.1082324).

42. Sharon Gilead, Haguy Wolfenson, Ehud Gazit, **Molecular Mapping of the Recognition Interface between the Islet Amyloid Polypeptide and Insulin**, Angewandte Chemie International Edition 2006 (doi: 10.1002/anie.200602034).

43. S. Giunta, M.B. Valli, R. Galeazzi, P. Fattoretti, E.H. Corder, L. Galeazzi, **Transthyretin inhibition of amyloid beta aggregation and toxicity**, Clinical Biochemistry 2005 (doi: 10.1016/j.clinbiochem.2005.08.007).

44. Sarah L Griner, Paul Seidler, Jeannette Bowler, Kevin A Murray, Tianxiao Peter Yang, Shruti Sahay, Michael R Sawaya, Duilio Cascio, Jose A Rodriguez, Stephan Philipp, Justyna Sosna, Charles G Glabe, Tamir Gonen, David S Eisenberg, **Structure-based inhibitors of amyloid beta core suggest a common interface with tau**, eLife 2019 (doi: 10.7554/elife.46924).

45. Lei Gu, Zhefeng Guo, **Alzheimer's A 42 and A 40 peptides form interlaced amyloid fibrils**, Journal of Neurochemistry 2013 (doi: 10.1111/jnc.12202).

46. Neal D. Hammer, Jens C. Schmidt, Matthew R. Chapman, **The curli nucleator protein, CsgB, contains an amyloidogenic domain that directs CsgA polymerization**, Proceedings of the National Academy of Sciences 2007 (doi: 10.1073/pnas.0703310104).

47. Xiuping Hao, Jie Zheng, Yan Sun, Xiaoyan Dong, **Seeding and Cross-Seeding Aggregations of A 40 and Its N-Terminal-Truncated Peptide A 11–40**, Langmuir 2019 (doi: 10.1021/acs.langmuir.8b03599).

48. Mamoru Haratake, Tohru Takiguchi, Naho Masuda, Sakura Yoshida, Takeshi Fuchigami, Morio Nakayama, **Amyloid formation characteristics of GNNQQNY from yeast prion protein Sup35 and its seeding with heterogeneous polypeptides**, Colloids and Surfaces B: Biointerfaces 2017 (doi: 10.1016/j.colsurfb.2016.10.011).

49. Kevin Hartman, Jeffrey R. Brender, Kazuaki Monde, Akira Ono, Margery L. Evans, Nataliya Popovych, Matthew R. Chapman, Ayyalusamy Ramamoorthy, **Bacterial curli protein promotes the conversion of PAP248-286into the amyloid SEVI: cross-seeding of dissimilar amyloid sequences**, PeerJ 2013 (doi: 10.7717/peerj.5).

50. Linda Helmfors, Andrea Boman, Livia Civitelli, Sangeeta Nath, Linnea Sandin, Camilla Janefjord, Heather McCann, Henrik Zetterberg, Kaj Blennow, Glenda Halliday, Ann-Christin Brorsson, Katarina Kågedal, **Protective properties of lysozyme on -amyloid pathology: implications for Alzheimer disease**, Neurobiology of Disease 2015 (doi: 10.1016/j.nbd.2015.08.024).

51. Chae Eun Heo, Tae Su Choi, Hugh I. Kim, **Competitive homo- and hetero- self-assembly of amyloid- 1–42 and 1–40 in the early stage of fibrillation**, International Journal of Mass Spectrometry 2018 (doi: 10.1016/j.ijms.2018.02.002).

52. Ryo Honda, **Amyloid- Peptide Induces Prion Protein Amyloid Formation: Evidence for Its Widespread Amyloidogenic Effect**, Angewandte Chemie International Edition 2018 (doi: 10.1002/anie.201800197).

53. Istvan Horvath, Pernilla Wittung-Stafshede, **Cross-talk between amyloidogenic proteins in type-2 diabetes and Parkinson's disease**, Proceedings of the National Academy of Sciences 2016 (doi: 10.1073/pnas.1610371113).

54. Istvan Horvath, Sandra Rocha, Pernilla Wittung-Stafshede, **In Vitro Analysis of -Synuclein Amyloid Formation and Cross-Reactivity**, Methods in Molecular Biology,Amyloid Proteins 2018 (doi: 10.1007/978-1-4939-7816-8_6).

55. Istvan Horvath, Igor A. Iashchishyn, Roman A. Moskalenko, Chao Wang, Sebastian K. T. S. Wärmländer, Cecilia Wallin, Astrid Gräslund, Gabor G. Kovacs, Ludmilla A. Morozova-Roche,

Co-aggregation of pro-inflammatory S100A9 with -synuclein in Parkinson's disease: ex vivo and in vitro studies, Journal of Neuroinflammation 2018 (doi: 10.1186/s12974-018-1210-9).

56. Yi-Hsuan Hsu, Yun-Wen Chen, Meng-Hsin Wu, Ling-Hsien Tu, **Protein Glycation by Glyoxal Promotes Amyloid Formation by Islet Amyloid Polypeptide**, Biophysical Journal 2019 (doi: 10.1016/j.bpj.2019.05.013).

57. Rundong Hu, Baiping Ren, Mingzhen Zhang, Hong Chen, Yonglan Liu, Lingyun Liu, Xiong Gong, Binbo Jiang, Jie Ma, Jie Zheng, **Seed-Induced Heterogeneous Cross-Seeding Self-Assembly of Human and Rat Islet Polypeptides**, ACS Omega 2017 (doi: 10.1021/acsomega.6b00559).

58. Rundong Hu, Mingzhen Zhang, Hong Chen, Binbo Jiang, Jie Zheng, **Cross-Seeding Interaction between -Amyloid and Human Islet Amyloid Polypeptide**, ACS Chemical Neuroscience 2015 (doi: 10.1021/acschemneuro.5b00192).

59. Rundong Hu, Mingzhen Zhang, Kunal Patel, Qiuming Wang, Yung Chang, Xiong Gong, Ge Zhang, Jie Zheng, **Cross-Sequence Interactions between Human and Rat Islet Amyloid Polypeptides**, Langmuir 2014 (doi: 10.1021/la500632d).

60. Alexandre I. Ilitchev, Maxwell J. Giammona, Carina Olivas, Sarah L. Claud, Kristi L. Lazar Cantrell, Chun Wu, Steven K. Buratto, Michael T. Bowers, **Hetero-oligomeric Amyloid Assembly and Mechanism: Prion Fragment PrP(106–126) Catalyzes the Islet Amyloid Polypeptide -Hairpin**, Journal of the American Chemical Society 2018 (doi: 10.1021/jacs.8b05925).

61. Yuji Inoue, Shigeko Kawai-Noma, Ayumi Koike-Takeshita, Hideki Taguchi, Masasuke Yoshida, **Yeast prion protein New1 can break Sup35 amyloid fibrils into fragments in an ATP-dependent manner**, Genes to Cells 2011 (doi: 10.1111/j.1365-2443.2011.01510.x).

62. Emma T. A. S. JAIKARAN, Melanie R. NILSSON, Anne CLARK, **Pancreatic beta-cell granule peptides form heteromolecular complexes which inhibit islet amyloid polypeptide fibril formation**, Biochemical Journal 2004 (doi: 10.1042/bj20030852).

63. Neha Jain, Jörgen Ådén, Kanna Nagamatsu, Margery L. Evans, Xinyi Li, Brennan McMichael, Magdalena I. Ivanova, Fredrik Almqvist, Joel N. Buxbaum, Matthew R. Chapman, **Inhibition of curli assembly and Escherichia coli biofilm formation by the human systemic amyloid precursor transthyretin**, Proceedings of the National Academy of Sciences 2017 (doi: 10.1073/pnas.1708805114).

64. Asad Jan, Ozgun Gokce, Ruth Luthi-Carter, Hilal A. Lashuel, **The Ratio of Monomeric to Aggregated Forms of A 40 and A 42 Is an Important Determinant of Amyloid- Aggregation, Fibrillogenesis, and Toxicity**, Journal of Biological Chemistry 2008 (doi: 10.1074/jbc.m803159200).

65. Ibrahim Javed, Zhenzhen Zhang, Jozef Adamcik, Nicholas Andrikopoulos, Yuhuan Li, Daniel E. Otzen, Sijie Lin, Raffaele Mezzenga, Thomas P. Davis, Feng Ding, Pu Chun Ke, **Accelerated Amyloid Beta Pathogenesis by Bacterial Amyloid FapC**, Advanced Science 2020 (doi: 10.1002/advs.202001299).

66. Theodoros K. Karamanos, Arnout P. Kalverda, Gary S. Thompson, Sheena E. Radford, **Visualization of Transient Protein-Protein Interactions that Promote or Inhibit Amyloid Assembly**, Molecular Cell 2014 (doi: 10.1016/j.molcel.2014.05.026).

67. Kathryn M. Keefer, Kevin C. Stein, Heather L. True, **Heterologous prion-forming proteins interact to cross-seed aggregation in Saccharomyces cerevisiae**, Scientific Reports 2017 (doi: 10.1038/s41598-017-05829-5).

68. J. Kim, L. Onstead, S. Randle, R. Price, L. Smithson, C. Zwizinski, D. W. Dickson, T. Golde, E. McGowan, **A 40 Inhibits Amyloid Deposition In Vivo**, Journal of Neuroscience 2007 (doi: 10.1523/jneurosci.4849-06.2007).

69. Radosveta P. Koldamova, Iliya M. Lefterov, Martina I. Lefterova, John S. Lazo, **Apolipoprotein A-I Directly Interacts with Amyloid Precursor Protein and Inhibits A Aggregation and Toxicity**, Biochemistry 2001 (doi: 10.1021/bi002186k).

70. Nadejda Koloteva-Levine, Liam D. Aubrey, Ricardo Marchante, Tracey J. Purton, Jennifer R. Hiscock, Mick F. Tuite, Wei-Feng Xue, **Amyloid particles facilitate surface-catalyzed cross-seeding by acting as promiscuous nanoparticles**, Proceedings of the National Academy of Sciences 2021 (doi: 10.1073/pnas.2104148118).

71. Janett Köppen, Anja Schulze, Lisa Machner, Michael Wermann, Rico Eichentopf, Max Guthardt, Angelika Hähnel, Jessica Klehm, Marie-Christin Kriegeskorte, Maike Hartlage-Rübsamen, Markus Morawski, Stephan von Hörsten, Hans-Ulrich Demuth, Steffen Roßner, Stephan Schilling, **Amyloid-Beta Peptides Trigger Aggregation of Alpha-Synuclein In Vitro**, Molecules 2020 (doi: 10.3390/molecules25030580).

72. Mark R.H. Krebs, Ludmilla A. Morozova-Roche, Katie Daniel, Carol V. Robinson, Christopher M. Dobson, **Observation of sequence specificity in the seeding of protein amyloid fibrils**, Protein Science 2004 (doi: 10.1110/ps.04707004).

73. Pascal Krotee, Sarah L. Griner, Michael R. Sawaya, Duilio Cascio, Jose A. Rodriguez, Dan Shi, Stephan Philipp, Kevin Murray, Lorena Saelices, Ji Lee, Paul Seidler, Charles G. Glabe, Lin Jiang, Tamir Gonen, David S. Eisenberg, **Common fibrillar spines of amyloid- and human islet amyloid polypeptide revealed by microelectron diffraction and structure-based inhibitors**, Journal of Biological Chemistry 2018 (doi: 10.1074/jbc.m117.806109).

74. Inna Kuperstein, Kerensa Broersen, Iryna Benilova, Jef Rozenski, Wim Jonckheere, Maja Debulpaep, Annelies Vandersteen, Ine Segers-Nolten, Kees Van Der Werf, Vinod Subramaniam, Dries Braeken, Geert Callewaert, Carmen Bartic, Rudi D'Hooge, Ivo Cristiano Martins, Frederic Rousseau, Joost Schymkowitz, Bart De Strooper, **Neurotoxicity of Alzheimer's disease A peptides is induced by small changes in the A 42 to A 40 ratio**, The EMBO Journal 2010 (doi: 10.1038/emboj.2010.211).

75. Jennifer L. Larson, Andrew D. Miranker, **The Mechanism of Insulin Action on Islet Amyloid Polypeptide Fiber Formation**, Journal of Molecular Biology 2004 (doi: 10.1016/j.jmb.2003.10.045).

76. Annika Larsson, Susanna Malmström, Per Westermark, **Signs of cross-seeding: aortic medin amyloid as a trigger for protein AA deposition**, Amyloid 2011 (doi: 10.3109/13506129.2011.630761).

77. X. Li, X. Zhang, A. R. A. Ladiwala, D. Du, J. K. Yadav, P. M. Tessier, P. E. Wright, J. W. Kelly, J. N. Buxbaum, **Mechanisms of Transthyretin Inhibition of -Amyloid Aggregation In Vitro**, Journal of Neuroscience 2013 (doi: 10.1523/jneurosci.2561-13.2013).

78. Peng Liu, Shuai Zhang, Mei-sha Chen, Qian Liu, Chenxuan Wang, Chen Wang, Yan-Mei Li, Flemming Besenbacher, Mingdong Dong, **Co-assembly of human islet amyloid polypeptide (hIAPP)/insulin**, Chem. Commun. 2012 (doi: 10.1039/c1cc14285b).

79. Chang-Wei Liu, Benoit I. Giasson, Karen A. Lewis, Virginia M. Lee, George N. DeMartino, Philip J. Thomas, **A Precipitating Role for Truncated -Synuclein and the Proteasome in -Synuclein Aggregation**, Journal of Biological Chemistry 2005 (doi: 10.1074/jbc.m501508200).

80. Lin Liu, Regina M. Murphy, **Kinetics of Inhibition of -Amyloid Aggregation by Transthyretin**, Biochemistry 2006 (doi: 10.1021/bi0618520).

81. Kaho Long, Thomas L. Williams, Brigita Urbanc, **Insulin Inhibits A 42 Aggregation and Prevents A 42-Induced Membrane Disruption**, Biochemistry 2019 (doi: 10.1021/acs.biochem.9b00696).

82. Jinxia Lu, Shengnan Zhang, Xiaojuan Ma, Chunyu Jia, Zhenying Liu, Chengan Huang, Cong Liu, Dan Li, **Structural basis of the interplay between -synuclein and Tau in regulating pathological amyloid aggregation**, Journal of Biological Chemistry 2020 (doi: 10.1074/jbc.ra119.012284).

83. Kelvin C. Luk, Dustin J. Covell, Victoria M. Kehm, Bin Zhang, Insung Y. Song, Matthew D. Byrne, Rose M. Pitkin, Samantha C. Decker, John Q. Trojanowski, Virginia M.-Y. Lee, **Molecular and Biological Compatibility with Host Alpha-Synuclein Influences Fibril Pathogenicity**, Cell Reports 2016 (doi: 10.1016/j.celrep.2016.08.053).

84. Jinghui Luo, Sebastian K.T.S. Wärmländer, Astrid Gräslund, Jan Pieter Abrahams, **Non-chaperone Proteins Can Inhibit Aggregation and Cytotoxicity of Alzheimer Amyloid Peptide**, Journal of Biological Chemistry 2014 (doi: 10.1074/jbc.m114.574947).

85. Jinghui Luo, Sebastian K. T. S. Wärmländer, Astrid Gräslund, Jan Pieter Abrahams, **Reciprocal Molecular Interactions between the A Peptide Linked to Alzheimer's Disease and Insulin Linked to Diabetes Mellitus Type II**, ACS Chemical Neuroscience 2016 (doi: 10.1021/acschemneuro.5b00325).

86. Jinghui Luo, Sebastian K. T. S. Wärmländer, Astrid Gräslund, Jan Pieter Abrahams, **Human lysozyme inhibits the in vitro aggregation of A peptides, which in vivo are associated with Alzheimer's disease**, Chemical Communications 2013 (doi: 10.1039/c3cc42325e).

87. Eliezer Masliah, Edward Rockenstein, Isaac Veinbergs, Yutaka Sagara, Margaret Mallory, Makoto Hashimoto, Lennart Mucke, **-Amyloid peptides enhance -synuclein accumulation and neuronal deficits in a transgenic mouse model linking Alzheimer's disease and Parkinson's disease**, Proceedings of the National Academy of Sciences 2001 (doi: 10.1073/pnas.211412398).

88. Fanling Meng, Daniel P. Raleigh, Andisheh Abedini, **Combination of Kinetically Selected Inhibitors in Trans Leads to Highly Effective Inhibition of Amyloid Formation**, Journal of the American Chemical Society 2010 (doi: 10.1021/ja1046186).

89. Chris T. Middleton, Peter Marek, Ping Cao, Chi-cheng Chiu, Sadanand Singh, Ann Marie Woys, Juan J. de Pablo, Daniel P. Raleigh, Martin T. Zanni, **Two-dimensional infrared spectroscopy reveals the complex behaviour of an amyloid fibril inhibitor**, Nature Chemistry 2012 (doi: 10.1038/nchem.1293).

90. Julijana Milojevic, Annie Raditsis, Giuseppe Melacini, **Human Serum Albumin Inhibits A Fibrillization through a "Monomer-Competitor" Mechanism**, Biophysical Journal 2009 (doi: 10.1016/j.bpj.2009.08.028).

91. Tarek Mohamed, Sarbjeet Singh Gujral, Praveen P. N. Rao, **Tau Derived Hexapeptide AcPHF6 Promotes Beta-Amyloid (A) Fibrillogenesis**, ACS Chemical Neuroscience 2017 (doi: 10.1021/acschemneuro.7b00433).

92. Fatemeh Mohammadi, Zeinab Takalloo, Hossein Rahmani, Mohammad Ali Nasiri Khalili, Khosro Khajeh, Gholamhossein Riazi, Reza H. Sajedi, **Interplay of isoform 1N4R tau protein and amyloid-peptide fragment 25–35 in reducing and non-reducing conditions**, The Journal of Biochemistry 2020 (doi: 10.1093/jb/mvaa101).

93. Marija Mucibabic, Pär Steneberg, Emmelie Lidh, Jurate Straseviciene, Agnieszka Ziolkowska, Ulf Dahl, Emma Lindahl, Helena Edlund, **-Synuclein promotes IAPP fibril formation in vitro and -cell amyloid formation in vivo in mice**, Scientific Reports 2020 (doi: 10.1038/s41598-020-77409-z).

94. Megan M. Murray, Summer L. Bernstein, Vy Nyugen, Margaret M. Condron, David B. Teplow, Michael T. Bowers, **Amyloid Protein: A 40 Inhibits A 42 Oligomerization**, Journal of the American Chemical Society 2009 (doi: 10.1021/ja8092604).

95. Charlotte Nerelius, Magnus Gustafsson, Kerstin Nordling, Annika Larsson, Jan Johansson, **Anti-Amyloid Activity of the C-Terminal Domain of proSP-C against Amyloid -Peptide and Medin**, Biochemistry 2009 (doi: 10.1021/bi900135c).

96. Krzysztof Nieznanski, Krystyna Surewicz, Shugui Chen, Hanna Nieznanska, Witold K. Surewicz, **Interaction between Prion Protein and A Amyloid Fibrils Revisited**, ACS Chemical Neuroscience 2014 (doi: 10.1021/cn500019c).

97. Krzysztof Nieznanski, Jin-Kyu Choi, Shugui Chen, Krystyna Surewicz, Witold K. Surewicz, **Soluble Prion Protein Inhibits Amyloid- (A) Fibrillization and Toxicity**, Journal of Biological Chemistry 2012 (doi: 10.1074/jbc.c112.400614).

98. Lina Nilsson, Annelie Pamrén, Tohidul Islam, Kristoffer Brännström, Solmaz A. Golchin, Nina Pettersson, Irina Iakovleva, Linda Sandblad, Anna L. Gharibyan, Anders Olofsson, **Transthyretin Interferes with A Amyloid Formation by Redirecting Oligomeric Nuclei into Non-Amyloid Aggregates**, Journal of Molecular Biology 2018 (doi: 10.1016/j.jmb.2018.06.005).

99. Bartosz Nizynski, Hanna Nieznanska, Robert Dec, Solomiia Boyko, Wojciech Dzwolak, Krzysztof Nieznanski, **Amyloidogenic cross-seeding of Tau protein: Transient emergence of structural variants of fibrils**, PLOS ONE 2018 (doi: 10.1371/journal.pone.0201182).

100. Justin M. Nussbaum, Stephan Schilling, Holger Cynis, Antonia Silva, Eric Swanson, Tanaporn Wangsanut, Kaycie Tayler, Brian Wiltgen, Asa Hatami, Raik Rönicke, Klaus Reymann, Birgit Hutter-Paier, Anca Alexandru, Wolfgang Jagla, Sigrid Graubner, Charles G. Glabe, Hans-Ulrich Demuth, George S. Bloom, **Prion-like behaviour and tau-dependent cytotoxicity of pyroglutamylated amyloid-** , Nature 2012 (doi: 10.1038/nature11060).

101. Takayuki Oikawa, Takashi Nonaka, Makoto Terada, Akira Tamaoka, Shin-ichi Hisanaga, Masato Hasegawa, **-Synuclein Fibrils Exhibit Gain of Toxic Function, Promoting Tau Aggregation and Inhibiting Microtubule Assembly**, Journal of Biological Chemistry 2016 (doi: 10.1074/jbc.m116.736355).

102. Kenjiro Ono, Ryoichi Takahashi, Tokuhei Ikeda, Masahito Yamada, **Cross-seeding effects of amyloid -protein and -synuclein**, Journal of Neurochemistry 2012 (doi: 10.1111/j.1471-4159.2012.07847.x).

103. Ofek Oren, Victor Banerjee, Ran Taube, Niv Papo, **An A 42 variant that inhibits intra- and extracellular amyloid aggregation and enhances cell viability**, Biochemical Journal 2018 (doi: 10.1042/bcj20180247).

104. Marie E. Oskarsson, Erik Hermansson, Ye Wang, Nils Welsh, Jenny Presto, Jan Johansson, Gunilla T. Westermark, **BRICHOS domain of Bri2 inhibits islet amyloid polypeptide (IAPP) fibril formation and toxicity in human beta cells**, Proceedings of the National Academy of Sciences 2018 (doi: 10.1073/pnas.1715951115).

105. Katiuscia Pagano, Denise Galante, Cristina D'Arrigo, Alessandro Corsaro, Mario Nizzari, Tullio Florio, Henriette Molinari, Simona Tomaselli, Laura Ragona, **Effects of Prion Protein on A 42 and Pyroglutamate-Modified A pE3-42 Oligomerization and Toxicity**, Molecular Neurobiology 2018 (doi: 10.1007/s12035-018-1202-x).

106. Bo Pang, Xiaoyu Zhuang, Xinyu Bian, Shu Liu, Zhiqiang Liu, Fengrui Song, **Studies on the cross-interaction between hIAPP and A 25-35 and the aggregation process in binary mixture by electrospray ionization-ion mobility-mass spectrometry**, Journal of Mass Spectrometry 2020 (doi: 10.1002/jms.4643).

107. Jonathan Pansieri, Igor A. Iashchishyn, Hussein Fakhouri, Lucija Ostojić, Mantas Malisauskas, Greta Musteikyte, Vytautas Smirnovas, Matthias M. Schneider, Tom Scheidt, Catherine K. Xu, Georg Meisl, Tuomas P. J. Knowles, Ehud Gazit, Rodolphe Antoine, Ludmilla A. Morozova-Roche, **Templating S100A9 amyloids on A fibrillar surfaces revealed by charge detection mass spectrometry, microscopy, kinetic and microfluidic analyses**, Chemical Science 2020 (doi: 10.1039/c9sc05905a).

108. Jonathan Pansieri, Lucija Ostojić, Igor A. Iashchishyn, Mazin Magzoub, Cecilia Wallin, Sebastian K.T.S. Wärmländer, Astrid Gräslund, Mai Nguyen Ngoc, Vytautas Smirnovas, Željko Svedružić, Ludmilla A. Morozova-Roche, **Pro-Inflammatory S100A9 Protein Aggregation Promoted by NCAM1 Peptide Constructs**, ACS Chemical Biology 2019 (doi: 10.1021/acschembio.9b00394).

109. Andréa C. Paula-Lima, M. Alejandra Tricerri, Jordano Brito-Moreira, Theresa R. Bomfim, Fabio F. Oliveira, Margaret H. Magdesian, Lea T. Grinberg, Rogerio Panizzutti, Sérgio T. Ferreira, **Human apolipoprotein A–I binds amyloid- and prevents A -induced neurotoxicity**, The International Journal of Biochemistry & Cell Biology 2009 (doi: 10.1016/j.biocel.2008.12.003).

110. Kris Pauwels, Thomas L. Williams, Kyle L. Morris, Wim Jonckheere, Annelies Vandersteen, Geoff Kelly, Joost Schymkowitz, Frederic Rousseau, Annalisa Pastore, Louise C. Serpell, Kerensa Broersen, **Structural Basis for Increased Toxicity of Pathological A 42:A 40 Ratios in Alzheimer Disease**, Journal of Biological Chemistry 2012 (doi: 10.1074/jbc.m111.264473).

111. Tyler J. Perlenfein, Jacob D. Mehlhoff, Regina M. Murphy, **Insights into the mechanism of cystatin C oligomer and amyloid formation and its interaction with -amyloid**, Journal of Biological Chemistry 2017 (doi: 10.1074/jbc.m117.786558).

112. Sergei Perov, Ofir Lidor, Nir Salinas, Nimrod Golan, Einav Tayeb- Fligelman, Maya Deshmukh, Dieter Willbold, Meytal Landau, **Structural Insights into Curli CsgA Cross- Fibril Architecture Inspire Repurposing of Anti-amyloid Compounds as Anti-biofilm Agents**, PLOS Pathogens 2019 (doi: 10.1371/journal.ppat.1007978).

113. Emily H. Pilkington, Yanting Xing, Bo Wang, Aleksandr Kakinen, Miaoyi Wang, Thomas P. Davis, Feng Ding, Pu Chun Ke, **Effects of Protein Corona on IAPP Amyloid Aggregation, Fibril Remodelling, and Cytotoxicity**, Scientific Reports 2017 (doi: 10.1038/s41598-017-02597-0).

114. Maa O. Quartey, Jennifer N. K. Nyarko, Jason M. Maley, Jocelyn R. Barnes, Maria A. C. Bolanos, Ryan M. Heistad, Kaeli J. Knudsen, Paul R. Pennington, Josef Buttigieg, Carlos E. De Carvalho, Scot C. Leary, Matthew P. Parsons, Darrell D. Mousseau, **The A (1–38) peptide is a negative regulator of the A (1–42) peptide implicated in Alzheimer disease progression**, Scientific Reports 2021 (doi: 10.1038/s41598-020-80164-w).

115. Lida Rahimi Araghi, Derek R. Dee, **Cross-Species and Cross-Polymorph Seeding of Lysozyme Amyloid Reveals a Dominant Polymorph**, Frontiers in Molecular Biosciences 2020 (doi: 10.3389/fmolb.2020.00206).

116. Kenneth B Rank, Adele M Pauley, Keshab Bhattacharya, Zhigang Wang, David B Evans, Timothy J Fleck, Jennifer A Johnston, Satish K Sharma, **Direct interaction of soluble human recombinant tau protein with A 1-42 results in tau aggregation and hyperphosphorylation by tau protein kinase II**, FEBS Letters 2002 (doi: 10.1016/s0014-5793(02)02376-1).

117. Jared K. Raynes, Li Day, Pauline Crepin, Mathew H. Horrocks, John A. Carver, **Coaggregation of -Casein and -Lactoglobulin Produces Morphologically Distinct Amyloid Fibrils**, Small 2017 (doi: 10.1002/smll.201603591).

118. Agata Rekas, Lucy Jankova, David C. Thorn, Roberto Cappai, John A. Carver, **Monitoring the prevention of amyloid fibril formation by -crystallin**, FEBS Journal 2007 (doi: 10.1111/j.1742-4658.2007.06144.x).

119. Agata Rekas, Christopher G Adda, J Andrew Aquilina, Kevin J Barnham, Margaret Sunde, Denise Galatis, Nicholas A Williamson, Colin L Masters, Robin F Anders, Carol V Robinson, Roberto Cappai, John A Carver, **Interaction of the Molecular Chaperone B-Crystallin with -Synuclein: Effects on Amyloid Fibril Formation and Chaperone Activity**, Journal of Molecular Biology 2004 (doi: 10.1016/j.jmb.2004.05.054).

120. Raimon Sabaté, Alba Espargaró, Natalia S. de Groot, Juan José Valle-Delgado, Xavier Fernàndez-Busquets, Salvador Ventura, **The Role of Protein Sequence and Amino Acid Composition in Amyloid Formation: Scrambling and Backward Reading of IAPP Amyloid Fibrils**, Journal of Molecular Biology 2010 (doi: 10.1016/j.jmb.2010.09.052).

121. Timothy R Sampson, Collin Challis, Neha Jain, Anastasiya Moiseyenko, Mark S Ladinsky, Gauri G Shastri, Taren Thron, Brittany D Needham, Istvan Horvath, Justine W Debelius, Stefan Janssen, Rob Knight, Pernilla Wittung-Stafshede, Viviana Gradinaru, Matthew Chapman, Sarkis K Mazmanian, **A gut bacterial amyloid promotes -synuclein aggregation and motor impairment in mice**, eLife 2020 (doi: 10.7554/elife.53111).

122. Hiromi M. Sanders, Robert Lust, Jan K. Teller, **Amyloid-beta peptide A p3-42 affects early aggregation of full-length A 1-42**, Peptides 2009 (doi: 10.1016/j.peptides.2009.01.027).

123. Puttur Santhoshkumar, Murugesan Raju, K. Krishna Sharma, **A-Crystallin Peptide 66SDRDKFVIFLDVKHF80 Accumulating in Aging Lens Impairs the Function of -Crystallin and Induces Lens Protein Aggregation**, PLoS ONE 2011 (doi: 10.1371/journal.pone.0019291).

124. Puttur Santhoshkumar, Krishna K. Sharma, **Inhibition of amyloid fibrillogenesis and toxicity by a peptide chaperone**, Molecular and Cellular Biochemistry 2004 (doi: 10.1023/b:mcbi.0000049373.15558.b8).

125. Magdalena Sastre, Miguel Calero, Monika Pawlik, Paul M Mathews, Asok Kumar, Vlatko Danilov, Stephen D Schmidt, Ralph A Nixon, Blas Frangione, Efrat Levy, **Binding of cystatin C to Alzheimer's amyloid inhibits in vitro amyloid fibril formation**, Neurobiology of Aging 2004 (doi: 10.1016/j.neurobiolaging.2003.11.006).

126. Anna Schimansky, Jay Kant Yadav, **Amyloid cross-sequence interaction between A (1-40) and A(66–80) in relation to the pathogenesis of cataract**, International Journal of Biological Macromolecules 2021 (doi: 10.1016/j.ijbiomac.2021.02.111).

127. Hamed Shaykhalishahi, Aziz Gauhar, Michael M. Wördehoff, Clara S. R. Grüning, Antonia N. Klein, Oliver Bannach, Matthias Stoldt, Dieter Willbold, Torleif Härd, Wolfgang Hoyer, **Contact between the 1 and 2 Segments of -Synuclein that Inhibits Amyloid Formation**, Angewandte Chemie International Edition 2015 (doi: 10.1002/anie.201503018).

128. Yao-Hsiang Shih, Ling-Hsien Tu, Ting-Yu Chang, Kiruthika Ganesan, Wei-Wei Chang, Pao-Sheng Chang, Yu-Sheng Fang, Yeh-Tung Lin, Lee-Way Jin, Yun-Ru Chen, **TDP-43 interacts with amyloid- , inhibits fibrillization, and worsens pathology in a model of Alzheimer's disease**, Nature Communications 2020 (doi: 10.1038/s41467-020-19786-7).

129. Maki Shirasaka, Kazuo Kuwata, Ryo Honda, **-Synuclein chaperone suppresses nucleation and amyloidogenesis of prion protein**, Biochemical and Biophysical Research Communications 2020 (doi: 10.1016/j.bbrc.2019.10.120).

130. Arshdeep Sidhu, Ine Segers-Nolten, Vinod Subramaniam, **Conformational Compatibility Is Essential for Heterologous Aggregation of -Synuclein**, ACS Chemical Neuroscience 2016 (doi: 10.1021/acschemneuro.5b00322).

131. Kalkena Sivanesam, Niels H. Andersen, **Inhibition of Human Amylin Amyloidogenesis by Human Amylin-Fragment Peptides: Exploring the Effects of Serine Residues and Oligomerization upon Inhibitory Potency**, Biochemistry 2017 (doi: 10.1021/acs.biochem.7b00739).

132. Tomas Sneideris, Mantas Ziaunys, Brett K.-Y. Chu, Rita P.-Y. Chen, Vytautas Smirnovas, **Self-Replication of Prion Protein Fragment 89-230 Amyloid Fibrils Accelerated by Prion Protein Fragment 107-143 Aggregates**, International Journal of Molecular Sciences 2020 (doi: 10.3390/ijms21197410).

133. Zachary A. Sorrentino, Niran Vijayaraghavan, Kimberly-Marie Gorion, Cara J. Riffe, Kevin H. Strang, Jason Caldwell, Benoit I. Giasson, **Physiological C-terminal truncation of -synuclein potentiates the prion-like formation of pathological inclusions**, Journal of Biological Chemistry 2018 (doi: 10.1074/jbc.ra118.005603).

134. Ewelina Stefaniak, Elena Atrian-Blasco, Wojciech Goch, Laurent Sabater, Christelle Hureau, Wojciech Bal, **The Aggregation Pattern of A 1–40 is Altered by the Presence of N -Truncated A 4–40 and/or Cu II in a Similar Way through Ionic Interactions**, Chemistry – A European Journal 2021 (doi: 10.1002/chem.202004484).

135. Weronika Surmacz-Chwedoruk, Hanna Nieznańska, Sławomir Wójcik, Wojciech Dzwolak, **Cross-Seeding of Fibrils from Two Types of Insulin Induces New Amyloid Strains**, Biochemistry 2012 (doi: 10.1021/bi301144d).

136. Olga Szczepankiewicz, Björn Linse, Georg Meisl, Eva Thulin, Birgitta Frohm, Carlo Sala Frigerio, Michael T. Colvin, Angela C. Jacavone, Robert G. Griffin, Tuomas Knowles, Dominic M. Walsh,

Sara Linse, **N-Terminal Extensions Retard A 42 Fibril Formation but Allow Cross-Seeding and Coaggregation with A 42**, Journal of the American Chemical Society 2015 (doi: 10.1021/jacs.5b07849).

137. Makoto Terada, Genjiro Suzuki, Takashi Nonaka, Fuyuki Kametani, Akira Tamaoka, Masato Hasegawa, **The effect of truncation on prion-like properties of -synuclein**, Journal of Biological Chemistry 2018 (doi: 10.1074/jbc.ra118.001862).

138. Joyce Tran, Dennis Chang, Frederick Hsu, Hongsu Wang, Zhefeng Guo, **Cross-seeding between A 40 and A 42 in Alzheimer's disease**, FEBS Letters 2016 (doi: 10.1002/1873-3468.12526).

139. Igor F. Tsigelny, Leslie Crews, Paula Desplats, Gideon M. Shaked, Yuriy Sharikov, Hideya Mizuno, Brian Spencer, Edward Rockenstein, Margarita Trejo, Oleksandr Platoshyn, Jason X.-J. Yuan, Eliezer Masliah, **Mechanisms of Hybrid Oligomer Formation in the Pathogenesis of Combined Alzheimer's and Parkinson's Diseases**, PLoS ONE 2008 (doi: 10.1371/journal.pone.0003135).

140. Jonathan Vaneyck, Ine Segers-Nolten, Kerensa Broersen, Mireille M.A.E. Claessens, **Cross-seeding of alpha-synuclein aggregation by amyloid fibrils of food proteins**, Journal of Biological Chemistry 2021 (doi: 10.1016/j.jbc.2021.100358).

141. David L Vanik, Krystyna A Surewicz, Witold K Surewicz, **Molecular Basis of Barriers for Interspecies Transmissibility of Mammalian Prions**, Molecular Cell 2004 (doi: 10.1016/s1097-2765(04)00155-8).

142. Bruno Vasconcelos, Ilie-Cosmin Stancu, Arjan Buist, Matthew Bird, Peng Wang, Alexandre Vanoosthuyse, Kristof Van Kolen, An Verheyen, Pascal Kienlen-Campard, Jean-Noël Octave, Peter Baatsen, Diederik Moechars, Ilse Dewachter, **Heterotypic seeding of Tau fibrillization by pre-aggregated Abeta provides potent seeds for prion-like seeding and propagation of Tau-pathology in vivo**, Acta Neuropathologica 2016 (doi: 10.1007/s00401-015-1525-x).

143. Yakov A. Vitrenko, Elena O. Gracheva, Janet E. Richmond, Susan W. Liebman, **Visualization of Aggregation of the Rnq1 Prion Domain and Cross-seeding Interactions with Sup35NM**, Journal of Biological Chemistry 2007 (doi: 10.1074/jbc.m609269200).

144. Cecilia Wallin, Yoshitaka Hiruma, Sebastian K. T. S. Wärmländer, Isabelle Huvent, Jüri Jarvet, Jan Pieter Abrahams, Astrid Gräslund, Guy Lippens, Jinghui Luo, **The Neuronal Tau Protein Blocks in Vitro Fibrillation of the Amyloid- (A) Peptide at the Oligomeric Stage**, Journal of the American Chemical Society 2018 (doi: 10.1021/jacs.7b13623).

145. Chao Wang, Alexey G. Klechikov, Anna L. Gharibyan, Sebastian K. T. S. Wärmländer, Jüri Jarvet, Lina Zhao, Xueen Jia, S. K. Shankar, Anders Olofsson, Thomas Brännström, Yuguang Mu, Astrid Gräslund, Ludmilla A. Morozova-Roche, **The role of pro-inflammatory S100A9 in Alzheimer's disease amyloid-neuroinflammatory cascade**, Acta Neuropathologica 2013 (doi: 10.1007/s00401-013-1208-4).

146. Hui Wang, Daniel P. Raleigh, **The Ability of Insulin To Inhibit the Formation of Amyloid by Pro-Islet Amyloid Polypeptide Processing Intermediates Is Significantly Reduced in the Presence of Sulfated Glycosaminoglycans**, Biochemistry 2014 (doi: 10.1021/bi4015488).

147. GuoZhen Wang, Alan R. Fersht, **Propagation of aggregated p53: Cross-reaction and coaggregation vs. seeding**, Proceedings of the National Academy of Sciences 2015 (doi: 10.1073/pnas.1500262112).

148. Sanduni Wasana Jayaweera, Solmaz Surano, Nina Pettersson, Elvira Oskarsson, Lovisa Lettius, Anna Gharibyan, Intissar Anan, Anders Olofsson, **Mechanisms of Transthyretin Inhibition of IAPP Amyloid Formation**, Biomolecules 2021 (doi: 10.3390/biom11030411).

149. Takahiro Watanabe-Nakayama, Maika Nawa, Hiroki Konno, Noriyuki Kodera, Toshio Ando, David B. Teplow, Kenjiro Ono, **Self- and Cross-Seeding on -Synuclein Fibril Growth Kinetics**

and Structure Observed by High-Speed Atomic Force Microscopy, ACS Nano 2020 (doi: 10.1021/acsnano.0c03074).

150. E. A. Waxman, B. I. Giasson, **Induction of Intracellular Tau Aggregation Is Promoted by -Synuclein Seeds and Provides Novel Insights into the Hyperphosphorylation of Tau**, Journal of Neuroscience 2011 (doi: 10.1523/jneurosci.0297-11.2011).

151. Tanja Weiffert, Georg Meisl, Patrick Flagmeier, Suman De, Christopher J. R. Dunning, Birgitta Frohm, Henrik Zetterberg, Kaj Blennow, Erik Portelius, David Klenerman, Christopher M. Dobson, Tuomas P. J. Knowles, Sara Linse, **Increased Secondary Nucleation Underlies Accelerated Aggregation of the Four-Residue N-Terminally Truncated A 42 Species A 5–42**, ACS Chemical Neuroscience 2019 (doi: 10.1021/acschemneuro.8b00676).

152. Tony Werner, Ranjeet Kumar, Istvan Horvath, Nathalie Scheers, Pernilla Wittung-Stafshede, **Abundant fish protein inhibits -synuclein amyloid formation**, Scientific Reports 2018 (doi: 10.1038/s41598-018-23850-0).

153. Gunilla T. Westermark, Per Westermark, **Transthyretin and Amyloid in the Islets of Langerhans in Type-2 Diabetes**, Experimental Diabetes Research 2008 (doi: 10.1155/2008/429274).

154. Hanna Willander, Jenny Presto, Glareh Askarieh, Henrik Biverstål, Birgitta Frohm, Stefan D. Knight, Jan Johansson, Sara Linse, **BRICHOS Domains Efficiently Delay Fibrillation of Amyloid -Peptide**, Journal of Biological Chemistry 2012 (doi: 10.1074/jbc.m112.393157).

155. Jonathan K. Williams, Xue Yang, Tamr B. Atieh, Michael P. Olson, Sagar D. Khare, Jean Baum, **Multi-Pronged Interactions Underlie Inhibition of -Synuclein Aggregation by -Synuclein**, Journal of Molecular Biology 2018 (doi: 10.1016/j.jmb.2018.05.024).

156. Hisashi Yagi, Eiko Kusaka, Kunihiro Hongo, Tomohiro Mizobata, Yasushi Kawata, **Amyloid Fibril Formation of -Synuclein Is Accelerated by Preformed Amyloid Seeds of Other Proteins**, Journal of Biological Chemistry 2005 (doi: 10.1074/jbc.m508623200).

157. Takahiro Yamaguchi, Katsumi Matsuzaki, Masaru Hoshino, **Interaction between soluble A -(1-40) monomer and A -(1-42) fibrils probed by paramagnetic relaxation enhancement**, FEBS Letters 2013 (doi: 10.1016/j.febslet.2013.02.008).

158. Li-Mei Yan, Aleksandra Velkova, Marianna Tatarek-Nossol, Erika Andreetto, Aphrodite Kapurniotu, **IAPP Mimic Blocks A Cytotoxic Self-Assembly: Cross-Suppression of Amyloid Toxicity of A and IAPP Suggests a Molecular Link between Alzheimer's Disease and Type II Diabetes**, Angewandte Chemie International Edition 2007 (doi: 10.1002/anie.200604056).

159. Li-Mei Yan, Marianna Tatarek-Nossol, Aleksandra Velkova, Athanasios Kazantzis, Aphrodite Kapurniotu, **Design of a mimic of nonamyloidogenic and bioactive human islet amyloid polypeptide (IAPP) as nanomolar affinity inhibitor of IAPP cytotoxic fibrillogenesis**, Proceedings of the National Academy of Sciences 2006 (doi: 10.1073/pnas.0507471103).

160. Li-Mei Yan, Aleksandra Velkova, Marianna Tatarek-Nossol, Gerhard Rammes, Andrei Sibaev, Erika Andreetto, Michael Kracklauer, Maria Bakou, Eleni Malideli, Burkhard Göke, Jörg Schirra, Martin Storr, Aphrodite Kapurniotu, **Selectively N-Methylated Soluble IAPP Mimics as Potent IAPP Receptor Agonists and Nanomolar Inhibitors of Cytotoxic Self-Assembly of Both IAPP and A 40**, Angewandte Chemie International Edition 2013 (doi: 10.1002/anie.201302840).

161. Motokuni Yonetani, Takashi Nonaka, Masami Masuda, Yuki Inukai, Takayuki Oikawa, Shin-ichi Hisanaga, Masato Hasegawa, **Conversion of Wild-type -Synuclein into Mutant-type Fibrils and Its Propagation in the Presence of A30P Mutant**, Journal of Biological Chemistry 2009 (doi: 10.1074/jbc.m807482200).

162. Brian K. Yoo, Yiling Xiao, Dan McElheny, Yoshitaka Ishii, **E22G Pathogenic Mutation of -Amyloid (A) Enhances Misfolding of A 40 by Unexpected Prion-like Cross Talk between A 42 and A 40**, Journal of the American Chemical Society 2018 (doi: 10.1021/jacs.7b13660).

163. Nadine D. Younan, Ko-Fan Chen, Ruth-Sarah Rose, Damian C. Crowther, John H. Viles, **Prion protein stabilizes amyloid- (A) oligomers and enhances A neurotoxicity in a Drosophila model of Alzheimer's disease**, Journal of Biological Chemistry 2018 (doi: 10.1074/jbc.ra118.003319).

164. Nadine D. Younan, Claire J. Sarell, Paul Davies, David R. Brown, John H. Viles, **The cellular prion protein traps Alzheimer's A in an oligomeric form and disassembles amyloid fibers**, The FASEB Journal 2013 (doi: 10.1096/fj.12-222588).

165. Lydia M. Young, Ling-Hsien Tu, Daniel P. Raleigh, Alison E. Ashcroft, Sheena E. Radford, **Understanding co-polymerization in amyloid formation by direct observation of mixed oligomers**, Chemical Science 2017 (doi: 10.1039/c7sc00620a).

166. Keisuke Yuzu, Naoki Yamamoto, Masahiro Noji, Masatomo So, Yuji Goto, Tetsushi Iwasaki, Motonari Tsubaki, Eri Chatani, **Multistep Changes in Amyloid Structure Induced by Cross-Seeding on a Rugged Energy Landscape**, Biophysical Journal 2021 (doi: 10.1016/j.bpj.2020.12.005).

167. Masihuz Zaman, Maria Andreasen, **Cross-talk between individual phenol-soluble modulins in Staphylococcus aureus biofilm enables rapid and efficient amyloid formation**, eLife 2020 (doi: 10.7554/elife.59776).

168. Ce Zhang, Yonggang Liu, Jonathan Gilthorpe, Johan R. C. van der Maarel, **MRP14 (S100A9) Protein Interacts with Alzheimer Beta-Amyloid Peptide and Induces Its Fibrillization**, PLoS ONE 2012 (doi: 10.1371/journal.pone.0032953).

169. Yanxian Zhang, Mingzhen Zhang, Yonglan Liu, Dong Zhang, Yijing Tang, Baiping Ren, Jie Zheng, **Dual amyloid cross-seeding reveals steric zipper-facilitated fibrillization and pathological links between protein misfolding diseases**, Journal of Materials Chemistry B 2021 (doi: 10.1039/d0tb02958k).

170. Li Na Zhao, Tong Zhang, Ce Zhang, Chao Wang, Ludmilla A. Morozova-Roche, Lock Yue Chew, Yuguang Mu, **S100A9 induces aggregation-prone conformation in Abeta peptides: a combined experimental and simulation study**, RSC Advances 2013 (doi: 10.1039/c3ra43665a).

171. Yizhou Zhou, Daniel Smith, Bryan J. Leong, Kristoffer Brännström, Fredrik Almqvist, Matthew R. Chapman, **Promiscuous Cross-seeding between Bacterial Amyloids Promotes Interspecies Biofilms**, Journal of Biological Chemistry 2012 (doi: 10.1074/jbc.m112.383737).

172. Mantas Ziaunys, Andrius Sakalauskas, Tomas Sneideris, Vytautas Smirnovas, **Lysozyme Fibrils Alter the Mechanism of Insulin Amyloid Aggregation**, International Journal of Molecular Sciences 2021 (doi: 10.3390/ijms22041775).
